# A PPARγ ligand present in Actinidia fruit (*Actinidia chrysantha*) is identified as dilinolenoyl galactosyl glycerol

**DOI:** 10.1042/BSR20120110

**Published:** 2013-05-15

**Authors:** Harry Martin, Tony K. McGhie, Rona C. M. Lunken

**Affiliations:** Food Innovation, the New Zealand Institute for Plant and Food Research Ltd, Private Bag 11 600, Palmerston North 4442, New Zealand

**Keywords:** chloroplast, dilinolenoylgalactosylglycerol, ligand, monogalactosyl diacylglycerol, PPARγ, slow-binding, DCM, dichloromethane, DGDG, digalactosyl diacylglycerol, DLGG, dilinolenoyl galactosyl glycerol, DOG, 1,3-dioleoylglycerol, FP, fluorescence polarization, LC–ESI–MS, liquid chromatography electrospray ionization mass spectrometry, MGDG, monogalactosyl diacylglycerol, mP, millipolarization, PG, prostaglandin, PKC, protein kinase C, PPARγ, peroxisome-proliferator-activated receptor γ

## Abstract

Activity-guided fractionation of Actinidia fruit species, including Kiwifruit, has identified DLGG (dilinolenoyl galactosyl glycerol) as a potent PPARγ (peroxisome-proliferator-activated receptor γ) ligand. DLGG is a type of MGDG (monogalactosyl diacylglycerol) and is present in all chloroplasts, and therefore all green fruits and vegetables. PPARγ is a ligand-activated transcription factor that regulates glucose metabolism and inflammation. An ethyl acetate extract of *Actinidia chrysantha* was fractionated by HPLC and the PPARγ-binding activity was detected by FP (fluorescence polarization). Linoleic and α-linolenic acids in *A. chrysantha* were readily detected as PPARγ ligands. Slow-binding PPARγ ligands were detected in several hydrophobic fractions. High-resolution MS identified DLGG as one of these ligands and confirmed that its binding is non-covalent. DLGG is a slow-binding PPARγ ligand with an IC_50_ of 1.64 μM, ±0.093 achieved after 45 min equilibration. DLGG is the first example of a form of DAG (diacylglycerol) that is a PPARγ ligand. In addition, DLGG is the first reported glycolipid ligand for PPARγ and also the first non-covalent, slow-binding PPARγ ligand.

## INTRODUCTION

PPARγ (peroxisome-proliferator-activated receptor γ) is a ligand-activated transcription factor and the target of the thiazolidinedione group of anti-diabetic drugs [1]. PPARγ is expressed in a wide variety of tissues [2]. In adipocytes, PPARγ activation increases lipogenesis and cell differentiation [3], whereas in muscle, PPARγ activation stimulates insulin sensitivity [4]. In lymphocytes [5] and macrophages [6], PPARγ agonists exert anti-inflammatory effects. PPARγ ligands may be natural and intrinsic [PG (prostaglandin) J2], natural and extrinsic e.g. dietary α-linolenic acid, or synthetic e.g. the drug rosiglitazone. Certain natural and synthetic ligands bind covalently to Cys^285^ in the PPARγ ligand-binding domain. These covalent ligands include PG J2 [7], unsaturated keto fatty acids [8] and the synthetic antagonist GW9662 [9].

Owing to its anti-diabetic and anti-inflammatory physiological role, the discovery of natural dietary PPARγ ligands and activators is of importance for human health and nutrition. The phytochemicals cyanidin-3-glucoside and protocatechuic acid are reported to up-regulate PPARγ in adipocytes [10]. PUFA (polyunsaturated fatty acids) activators of PPARγ include *n*−3 fatty acids such as α-linolenic acid, docosahexaenoic and eicosapentaenoic acid [11]. The health benefits of dietary sources of PPARγ activators have been reviewed recently [12].

Actinidia species were selected as a potential source of new PPARγ ligands not only because they are rich in α-linolenic acid, a well-known PPARγ ligand, but also because they contain a variety of polyunsaturated compounds such as lutein, β-carotene and various other carotenoids [13]. The *Actinidia deliciosa* species, better known as kiwifruit, is very common in the human diet yet several other Actinidia species are cultivated as a human food source or have been long-used in traditional remedies (e.g. *Actinidia eriantha* and *Actinidia polygama*) [14–19].

α-Linolenic acid is often a component of galactolipids such as MGDG (monogalactosyl diacylglycerols) and DGDG (di-galactosyl diacylglycerols). MGDG and DGDG are abundant in the chloroplasts of plants and in the light harvesting plastids of cyanobacteria. In plants, their concentrations increase to maintain membrane fluidity as an adaptation to increasing light intensity [20]. In wheat exposed to strong light, α-linolenic acid represents approximately 75% percent of total chloroplast MGDG [20]. The MGDG content of fruits and vegetable vary according to their chloroplast content; by wet weight, broccoli, kiwifruit and lemons contain approximately 350, 55 and 6 mg/kg, respectively [21].

In this study, we have used activity guided fractionation to identify a new PPARγ ligand in ethyl acetate extracts of Actinidia species, of which kiwifruit (*A. deliciosa*) is a member. Our interest has focused on DLGG (dilinolenoyl galactosyl glycerol) which is a specific type of MGDG in which the acyl groups are linolenic acid. PPARγ-binding activity was measured using a FP (fluorescence polarization) assay, and the active molecule was characterized using HPLC and MS.

## EXPERIMENTAL

### Chemicals and materials

Silica gel (30–70 μm, 60 Å) was purchased from Grace Davison Discovery Sciences. Troglitazone, GW9662, DOG (1,3-dioleoylglycerol) and PG (prostaglandin) J2 were supplied by Sigma–Aldrich. α-linolenic acid and linoleic acid were from Analabs Inc. and MGDG containing DLGG was supplied by Larodan [product 59-1200]. LC-MS grade acetonitrile was from Fischer Scientific, methanol (ChromAR) was from Mallinckrodt Chemicals, and ethanol (95%) was from LabServ.

### Plant material and ethyl acetate extraction of Actinidia fruits

Actinidia species were grown in the Plant & Food Research orchard and the fruit harvested when ripe. Fruit were freeze-dried, pulverized, extracted for 1 h at room temperature (22°C) in ethyl acetate (10 ml solvent: 1 g freeze-dried powder), filtered and freeze-dried again to remove solvent. The freeze-dried extract was then dissolved in DMSO at a ratio of 1 ml DMSO: 0.5 g original dry fruit equivalent). The Actinidia species used were: *Actinidia chinensis*, *A. polygama*, *A. eriantha*, *Actinidia glaucophylla*, *A. chrysantha*, *A. deliciosa* (unripe), *A. deliciosa* (ripe). Each extract was diluted serially in DMSO and assayed for PPARγ-binding activity using FP.

### FP assay of PPARγ ligands

FP assays were performed on the Tecan Safire2 fluorescence microplate reader (Tecan) at 22°C, in a volume of 20 μl in Nunc 384-well black, shallow microplates. The PPARγ (green) competitive binding assay (PolarScreen™) kit was supplied by Invitrogen Corporation. For measurement of FP, λ_ex_ and λ_em_ were set at 470 and 525 nm. Samples in DMSO were added to the preformed ligand:receptor complex such that the final DMSO concentration was 1%. Polarization is expressed in mP (millipolarization) units. The FP technique depends on the fact that small fluorescent ligand bound to a receptor has a slow rotation and therefore emits highly polarized light. As the fluorescent ligand is displaced from the PPARγ receptor by a non-fluorescent competitor, the polarization (mP) value decreases due to the more rapid rotation of the unbound, low molecular mass, fluorescent ligand.

### Fraction preparation

Freeze-dried fruit of *A. chrysantha* were ground to a fine powder in a mortar and pestle and 50 g mixed with 400 ml of DCM (dichloromethane) and allowed to stand overnight at room temperature. The DCM was decanted, and the fruit residue extracted with a further 250 ml DCM. After decanting this both DCM extracts were combined, filtered through Whatman 3 filter paper and evaporated to dryness under vacuum at 50°C using a rotary evaporator. The residue was dissolved in 15 ml DCM and stored at 4°C until used. This extract was called AgDCM1. For fractionation by HPLC, 1 ml of AgDCM1 was evaporated to dryness under N_2_ and the residue dissolved in 2 ml MeOH. The HPLC system was composed of a Waters 2690 Solvent Delivery System with a Waters 996 diode array detector connected to a Foxy Junior (Isco) fraction collector all controlled by Chromeleon Chromatography Management System V6.8 (Thermo Dionex). The separation column was a Gemini 5 μm C18 4.6×250 mm (Phenomenex) and a binary solvent system was used with solvent A, MilliQ water and solvent B, acetonitrile. The mobile gradient was as follows: 10% A, 90% B, 0–0.5 min; linear gradient to 100% B, 0.5–15 min; composition held at 100% B, 15–34 min; return to initial conditions, 34–35 min. The chromatogram at 205 nm was used for peak detection and peaks were automatically collected into a 1 ml 96-well plate between 2–34 min.

### Semi-preparative HPLC

To isolate specific compounds the sample of AgDCM1 was fractionated using semi-preparative HPLC. The HPLC system was the same as that used above; however, the separation column was a Synergi Hydro 4 μm 10× 250 mm (Phenomenex) at a flow rate of 3.0 ml/min. The same solvents were used but the mobile gradient was as follows: 10% A, 90% B, 0–0.5 min; linear gradient to 100% B, 0.5–15 min; composition held at 100% B, 15-40 min; return to initial conditions, 40–41 min. Fractions were collected into 10 ml glass tubes and like fractions from individual injections were combined and evaporated to dryness.

### LC-QTOF-HRMS

The LC-MS system was composed of a Dionex Ultimate® 3000 Rapid Separation LC system and a micrOTOF QII mass spectrometer (Bruker Daltonics) and was operating in a positive mode with an ESI source. The LC system contained a SRD-3400 solvent rack/degasser, HPR-3400RS binary pump, WPS-3000RS thermostated autosampler and a TCC-3000RS thermostated column compartment. The analytical column was a Zorbax™ SB-C18 2.1×150 mm, 1.8 μm (Agilent) maintained at 50°C.

### Protein LC-HRMS

The molecular masses of PPARγ, and possible PPARγ conjugates, were measured by LC-ESI-HRMS. HPLC solvents were A=0.5% formic acid, and B=100% acetronitrile at a flow rate of 400 μl/min. The gradient was: 0–1 min, isocratic at 50% A, 50% B; 1–12 min, linear gradient to 100% B; 12–15 min, isocratic at 100% B; 15–16 min, linear gradient to 50% A, 50% B; to return to the initial conditions before another sample injection at 20 min. The injection volume for samples and standards was 5 μl. The micrOTOF QII was in a positive ion mode and the source parameters were: temperature 200°C; drying N_2_ flow 8 litres/min; nebulizer N_2_ 4.0 bar, endplate offset −500 V, capillary voltage 4000 V; mass range 100–1500 Da, acquired at two scans per second. Post-acquisition internal mass calibration used sodium formate clusters with the sodium formate delivered by a syringe pump at the start of each chromatographic analysis. Mass spectra were averaged across the protein HPLC peak and the molecular masses of PPARγ and the PPARγ conjugates were calculated using the ‘deconvolute’ function of DataAnalysis (Bruker Daltonics).

## RESULTS

### Survey of Actinidia species for PPARγ ligand activity

**Figure 1:**
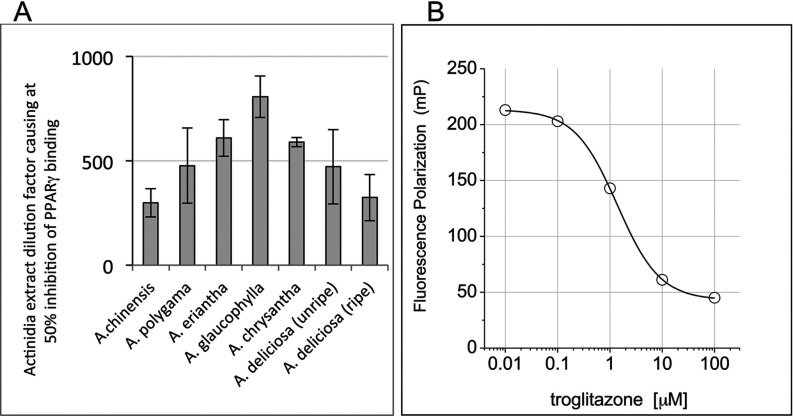
PPARγ binding activity in Actinidia extracts (**A**) PPARγ binding activity in the ethyl acetate extracts of a panel of Actinidia species fruits measured by FP. (**B**) Troglitazone dose–response curve demonstrating the validity of the FP assay.

PPARγ-binding activity was detected in all extracts and the activity varied among the different Actinidia species (Figure 1A). The strongest binding activity was observed with *A. glaucophylla* which has a PPARγ IC_50_ of 807±99.6. Figure 1(B) shows the dose–response curve for the reversible PPARγ ligand Troglitazone to validate the PPARγ FP assay. Due to the availability of a large quantity of *A. chrysantha* and because it also had abundant PPARγ-binding activity, this species was chosen as a source of starting material for the isolation of PPARγ-binding ligands in fruit of the Actinidia genus. A further extract of *A. chrysantha* was prepared and fractionated for compound identification.

### Fractionation of PPARγ-binding activity in *A. chrysantha* extract

**Figure 2:**
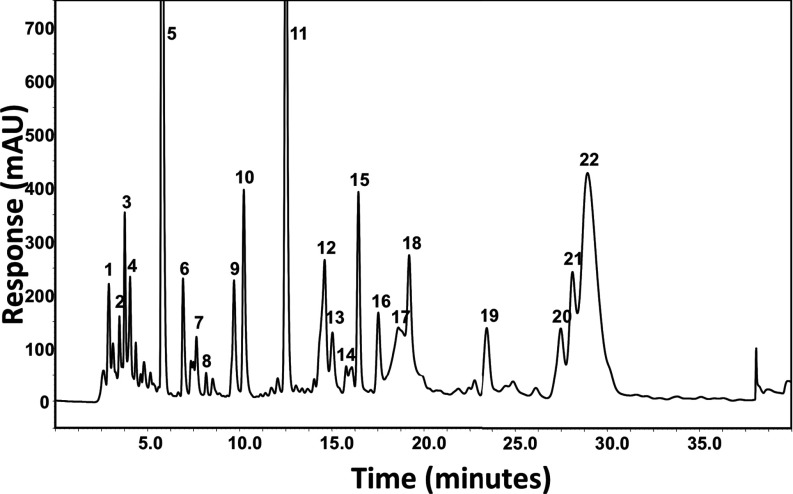
HPLC trace of the ethyl acetate extract of *A. chrysantha* HPLC fractions are numbered for subsequent analysis for PPARγ binding activity.

**Figure 3:**
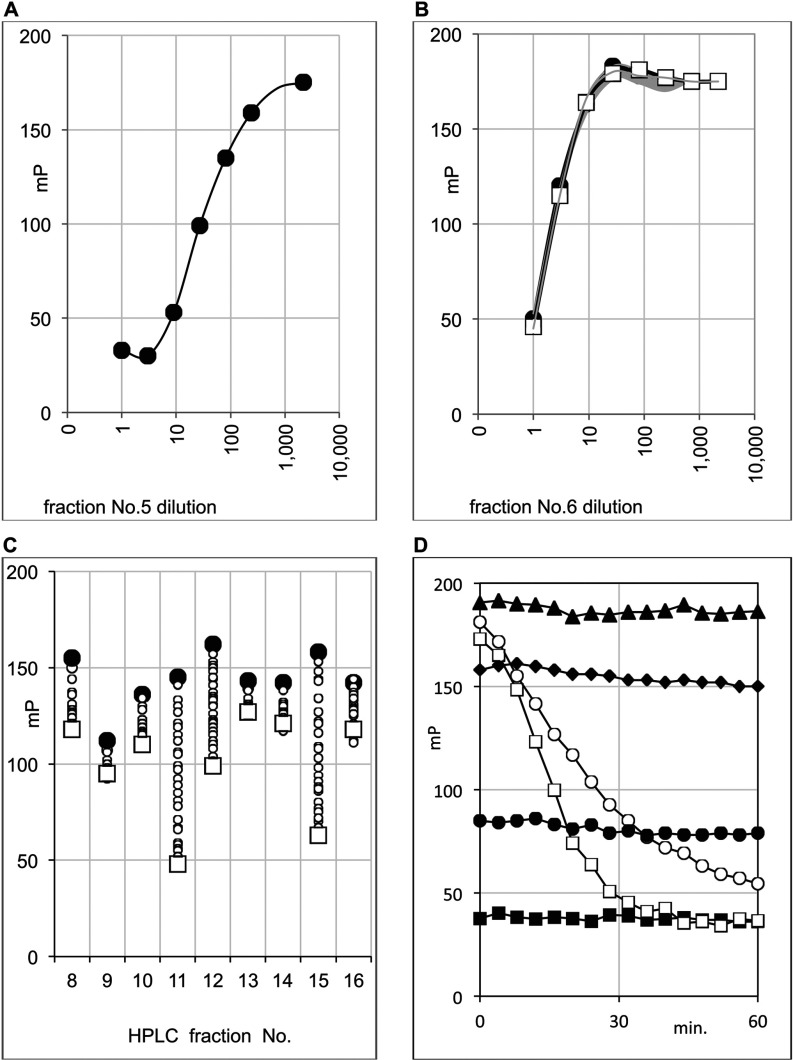
Fast and slow equilibrating PPARγ ligands (**A**–**C**) FP analysis of selected fractions from Figure 2 (*A. chrysantha*) for PPARγ binding activity. (**D**) HPLC fraction 11 comparing Troglitazone and GW9662 in PPARγ FP assay. (**A**) HPLC fraction 5 was tested in a 3-fold dilution series. (**B**) HPLC fraction 6 was tested in a 3-fold dilution series over a 60 min time course. Intermediate readings taken between the first and last readings are shown as faint grey lines. (**C**) HPLC fractions 8–16 were tested over a time course of 0 (●)–60 (□) min. Intermediate readings taken between the first and last readings are shown as small open circles. (**D**) 25 μM Troglitazone ■; 2.5 μM Troglitazone ●; 0.25 μM Troglitazone ◆; no ligand ▲; 25 nM GW9662 ○; HPLC fraction 11 □.

A DCM extract of *A. chrysantha* was prepared and RP (reverse—phase)-HPLC used to isolate factions that were tested for PPARγ-binding activity. Figure 2 shows the HPLC trace of the *A. chrysantha* extract. HPLC fractions showing PPARγ-binding activity were then selected and assayed in more detail in a dilution series and a time course assay (Figure 3). HPLC fractions 5 (Figure 3A) and 6 (Figure 3B) were tested at a top concentration of 1:40 and also in a 3-fold dilution series of this concentration, whereas the remaining fractions were tested only at a 1:40 final dilution of the sample. The data indicate the presence of two fast-equilibrating PPARγ ligands (Figures 3A and 3B). More lipophilic compounds with slow binding characteristics are evident in fractions 11, 12 and 15. The slow binding nature of the ligands in these fractions is evident since the mP value steadily decreases over a period of 60 min; the small circles in the Figure represent readings taken at 2 min intervals. For comparison, the constant value of the fast-equilibrating ligand in fraction 6 is shown by a series of overlapping grey lines.

Based on the UV/Vis spectral properties (results not shown) compounds 5 and 6 appeared to be fatty acids and with the aid of authentic standards and comparison of HPLC retention times and UV spectral properties, compound 5 was identified as α-linolenic acid and compound 6 as a linoleic acid. Both α-linolenic acid and linoleic acid have previously been reported as PPARγ activators [22–24]. As shown in Figure 3, α-linolenic acid and linoleic acid bind to PPARγ immediately under the assay conditions used. In comparison compounds 11, 12 and 15 show time-dependent binding to PPARγ (Figure 3C) as indicated by a reduction in mP over time. This behaviour suggests that these ligands might be binding covalently, whereas α-linolenic acid and linoleic acids bind non-covalently. The time-course binding curve for the covalent PPARγ ligand GW9662 at low concentrations is very similar to the slow binding curve of compound 11 (Figure 3D). By contrast, the rapidly equilibrating, reversible drug, Troglitazone shows stable equilibria at each concentration used ranging from 25 to 0.25 μM.

### Chemical identification of compound 11 using LC–ESI–MS (liquid chromatography electrospray ionization mass spectrometry)

**Figure 4:**
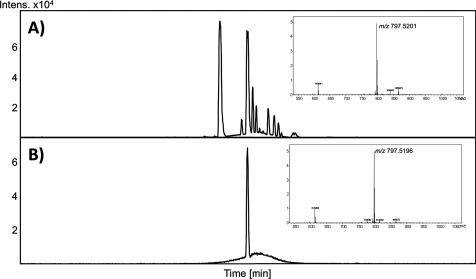
LC/MS chromatograms of (A) commercial standard containing DLGG, and (B) compound 11

Semi-preparative HPLC was used to isolate compound 11 and using direct introduction ESI-HRMS the positive and negative mass spectra were obtained. An intense positive ion at *m/z* 797.5163 was observed and is consistent with the elemental formula C_45_H_74_O_10_Na. In the negative ion spectra, two ion clusters were observed at *m/z* 809.5013 and *m/z* 819.5313. These ions correspond to elemental compositions of C_45_H_74_O_10_Cl and C_46_H_75_O_12_ respectively. These data suggest that compound 11 had an elemental composition of C_45_H_74_O_10_ and a molecular mass of 775.5355 Da. Furthermore, the daughter ion spectrum of *m/z* 809.5013 gave an ion at *m/z* 277.2187 which is consistent with linolenic acid. These data suggest that compound 11 is a glycolipid and the mass of the ions are consistent with an MGDG containing two linolenic acid moieties. To confirm this identification, an authentic standard was purchased and compared with compound 11 using LC–ESI–MS. The results are shown in Figure 4 and the identity of compound 11 as DLGG is confirmed both by HPLC retention times and high resolution mass spectral data.

### Direct binding to PPARγ using ESI-MS

Slow binding enzyme and receptor ligands are frequently covalent binders [25]. An example of a covalent binding PPARγ ligand (GW9662) is shown in Figure 3(D). The slow-binding behaviour of DLGG (compound 11) indicates that DLGG might bind covalently to PPARγ. ESI-HRMS of PPARγ was used to determine whether DLGG binds covalently to PPARγ and the results are shown in Table 1. The molecular mass of the PPARγ purchased from Invitrogen was shown to be 35919 Da with a further major component of 36096 Da. This is consistent with the amino acid composition of PPARγ. When IAF (5-iodoacetamidofluorescein) was added to PPARγ, the molecular mass of PPARγ increased by 389 Da, consistent with covalent modification of cysteine in the binding site of PPARγ. Similarly, when the known covalent PPARγ binder, GW9662 [9], is added, the molecular mass of PPARγ increases by 241 Da indicating that covalent binding has occurred. However, when DLGG was added to PPARγ the molecular mass of PPARγ did not increase indicating that DLGG does not bind covalently to PPARγ.

**Table 1:** Molecular masses of PPARγ and PPARγ conjugates as measured by ESI–MS Molecular mass of the PPARγ used is 35.9 kDa as specified by Invitrogen. The molecular mass of IAF is 515 Da, which results in the addition of 388 Da to a thiol when reaction occurs. The molecular mass of GW9662 is 276 Da, which results in the addition of 241 Da to a thiol when the reaction occurs.

Receptor	Ligand	Measured mass (kDa)
PPARγ	–	35.919
PPARγ	IAF	36.308
PPARγ	GW9662 (2-chloro-5-nitro-*N*-phenylbenzamide)	36.160
PPARγ	DLGG	35.919

### DOG is not a PPARγ ligand

**Figure 5:**
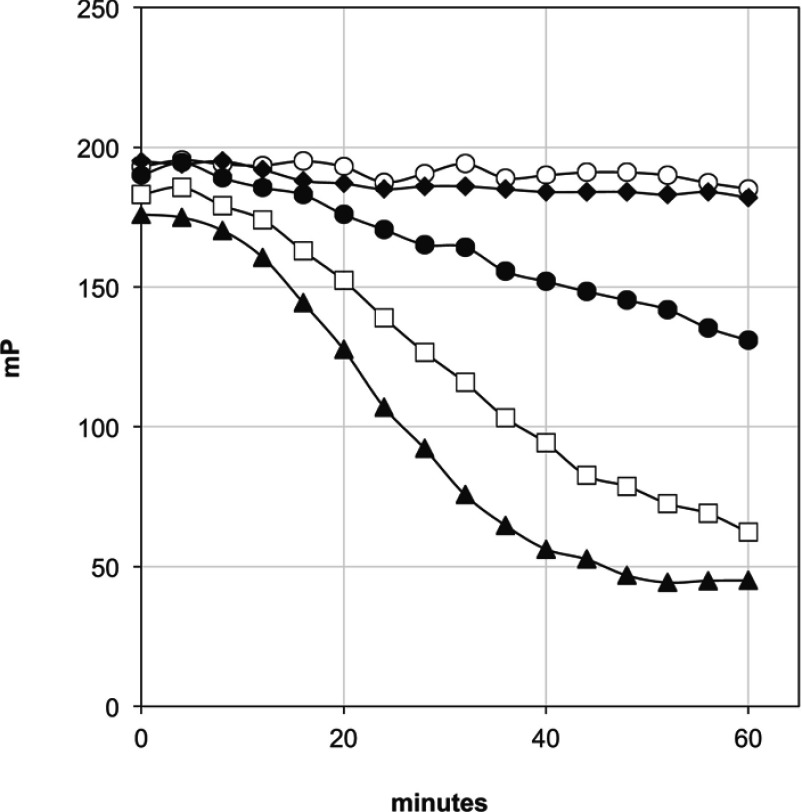
Comparison of DLGG with DOG for PPARγ binding Ligand concentration: 10 μM DLGG ▲; 3.3 μM DLGG □; 1.1 μM DLGG●; 100 μM DOG ◆; DMSO control ○.

To determine whether the slow binding of DLGG could also be demonstrated with another DAG (diacylglycerol), DOG was assayed in the same manner. A dilution series of DLGG and DOG in DMSO was tested in the PPARγ FP assay. The result (Figure 5) confirmed that DOG does not act as a PPARγ ligand. Although the DOG was tested at the same concentrations as DLGG, only the top concentration is shown in Figure 5 because none of the DOG data showed evidence of binding and the DOG curves overlapped.

The formal possibility exists that the slow-binding phenomenon is due to the slow release of non-esterified fatty acids during the assay due to contamination of the assay reagents with lipase activity. Conceivably the recombinant, bacterially derived PPARγ protein might contain traces of lipase. The addition of pancreatic or *Candida rugosa* lipase to an emulsion of triolein leads to the time- and lipase-dependent release of oleic acid which displaces the FP assay ligand (results not shown) in a manner identical with the slow-binding curves of DLGG in Figures 3 and 5. However, Figure 5 confirms that no oleic acid is produced from DOG during the assay, indicating that the DLGG slow-binding phenomenon is not due to a contamination of the recombinant PPARγ protein with lipase. Thus the apparent slow binding of DLGG to PPARγ is not due to the accidental production of linolenic acid during the FP assay.

## DISCUSSION

The finding that DLGG is a ligand for PPARγ is relevant for human health because DLGG and other MGDGs are abundant in the human diet. As a chloroplast component, MGDGs are present in all green (chlorophyll-containing) fruits and vegetables. Although we have isolated DLGG from Actinidia species, the highest concentrations of MGDG are in green vegetables, for example, parsley (*Petroselinum crispum*) is estimated to contain 184 mg MGDG/100 g wet weight, in comparison with 4.5–6.5 mg/100 g kiwifruit (*A. chinensis*) and 0.8 mg/100 g banana (*Musa manzano*) [21].

The time-course binding curve of DLGG is similar to that of GW9662, at low concentrations (results not shown). However, the mechanism underlying the slow binding of these two ligands appears to be quite different. The slow binding of GW9662 is due to the extremely low concentration of the compound in the assay. At low nanomolar concentrations of GW9662 the on-rate of binding is essentially diffusion controlled and GW9662 forms a covalent bond with PPARγ Cys285 [9]. By contrast, DLGG does not bind covalently to PPARγ (Table 1) and the slow binding of DLGG occurs in the low micromolar concentration range. The slow binding may indicate the ligand exists in a variety of conformations in the sample and only occasionally adopts a conformation which permits binding to the PPARγ. For example, if the assay concentration of DLGG is higher than its critical micelle concentration, the proportion of free DLGG in solution may be much lower than described in the Figure. Although the critical micelle concentration for DLGG is unknown to us, the critical micelle concentration of related plant lipid molecules is reported to be 13.1 μM for DGDG, 6.3 μM for sulphoquinovosyl DAG and 5.1 μM for DAG trimethylhomoserine [26]. These critical micelle concentration values are close to the DLGG concentrations which exhibit slow-binding to PPARγ and therefore suggest the true DLGG concentration in solution may be lower than the values reported in Figure 5. Another possible explanation for the slow binding of DLGG to PPARγ may be that the PPARγ conformation changes to accommodate DLGG in its binding site, i.e. DLGG may bind by an ‘induced-fit’ mechanism. Equilibration times of several hours have been observed when a peptide ligand binds to MHC class II molecules [27] by an induced-fit mechanism.

The physiological significance of DLGG–PPARγ interaction is unknown and it has not been confirmed in these experiments that DLGG can enter cells. Arguably the action of gut lipases might hydrolyse DLGG to release α-linolenic acid and glycerol before it is able to access gut epithelial cells. However, DLGG is reported to induce anti-inflammatory gene expression in chondrocytes in tissue culture [28]. In that study COX-2 (cyclooxygenase-2) is hypothesized to be the target of DLGG action. We would suggest that some anti-inflammatory activities of DLGG may stem from its binding to PPARγ.

DLGG is a galactose-containing analogue of DAG. DAG is a second messenger in PKC (protein kinase C) activation and PKC-mediated signal transduction. DAG recruits PKC to the internal surface of the plasma membrane and weakens the inhibitory pseudosubstrate domains control of the PKC catalytic domain [29]. Thus, the binding of DLGG, a glycosylated form of DAG, to PPARγ raises the possibility that PKC might compete with PPARγ for binding to limiting concentrations of DAG. DOG is a PKC activating co-factor [30] and is clearly not a PPARγ ligand (Figure 5). However, it is conceivable that 1,2-dioleoylglycerol or 1,2-dilinolenoyl glycerol might show some affinity for PPARγ. Whether the DLGG binding to PPARγ is dependent on the presence of galactose remains to be determined.

The slow-binding of DLGG to PPARγ is interesting because of DLGG's abundance in the human diet. It is tempting to speculate that the slow-binding phenomenon may also occur with intrinsic PPARγ ligands and might even suggest some regulatory role for PPARγ in which lipophilic second messengers may be gradually sequestered. Further characterization of the additional PPARγ binding components present in chloroplast containing foods is warranted. Although we have not demonstrated the physiological effects of DLGG on cells in terms of PPARγ dependent gene-expression, this is the first demonstration of a non-covalent, slow-binding PPARγ ligand. In addition, this is the first evidence that PPARγ can accommodate either DAG-type or glycolipid ligands.
